# Effects of supplemental feeding of Chinese herbal mixtures to perinatal sows on reproductive performance, immunity, and breast milk quality of sows

**DOI:** 10.3389/fvets.2024.1445216

**Published:** 2024-12-06

**Authors:** Xuelei Duan, Xiao Wang, Zhaonian Li, Chenggong Liu, Lu Zhang, Yongzhan Bao, Wanyu Shi, Xinghua Zhao

**Affiliations:** ^1^College of Traditional Chinese Veterinary Medicine, Hebei Agricultural University, Baoding, China; ^2^Hebei Provincial Veterinary Biotechnology Innovation Center, Baoding, China; ^3^Hebei Provincial Traditional Chinese Veterinary Medicine Technology Innovation Center, Baoding, China

**Keywords:** Chinese herbal mixtures, reproductive performance, immunity, breast milk, sows

## Abstract

The aim of this study was to investigate the impact of supplementary feeding with Chinese herbal mixtures on perinatal sows, focusing on their reproductive performance, immunity and breast milk quality. Sixty healthy pregnant sows (Large white, 4 parities) were randomly allocated into five treatment groups (*n* = 12 per group): the control group received a basal diet, the TRT1 group received a basal diet supplemented with 2 kg/t Bazhen powder (BZP), while the TRT2, TRT3, and TRT4 groups received a basal diet supplemented with 1 kg/t, 2 kg/t, and 3 kg/t Qi-Zhu-Gui-Shao soothing liver and replenishing blood powder (QZGSP), respectively. The trial lasted for a duration of 5 weeks, commencing from day 100 of gestation and concluding on day 21 postpartum. The results showed that supplemental feeding of 2 kg/t and 3 kg/t QZGSP to periparturient sows significantly improved reproductive performance to different degrees, as evidenced by the shortened farrowing intervals and increased average daily feed intake and milk yield. Supplemental feeding of 2 kg/t and/or 3 kg/t QZGSP significantly elevated levels of IL-4, IL-10, IgG, and IgA in sow serum while reduced levels of TNF-α and IL-1β in sow serum. In addition, supplemental feeding of 2 kg/t and 3 kg/t QZGSP to perinatal sows significantly increased the protein and fat content in colostrum and milk. Analysis of 16S rRNA gene amplicon sequencing data in colostrum and milk microbiota revealed that supplemental feeding of QZGSP to perinatal sows is influenced the composition of colostrum and milk composition in sows. Specifically, at the genus level, a decrease in the relative abundance of *Escherichia-Shigella*, *Staphylococcus* and *Streptococcus* was observed in the TRT3 and/or TRT4 groups on day 0 of lactation. The findings from this study indicate that supplemental feeding of 2 kg/t and 3 kg/t QZGSP significantly improved the reproductive performance, immunity and milk quality in sows. Therefore, QZGSP is a beneficial feed additive for perinatal sows.

## 1 Introduction

As we all know, sows play a pivotal role in modern intensive pig production conditions, and the health status and reproductive performance of sows are closely related to the production efficiency of pig enterprises. The health condition of sows before and after farrowing, as well as their lactation ability, directly affect the quantity and quality of colostrum and milk. This, in turn, affects the growth and development of piglets and their resistance to disease (1, 2). However, during late pregnancy and lactation periods, sows are frequently subjected to various stressors such as repeated services, environmental factors, and physiological changes etc. (3–5), which decrease their reproductive performance, lactation capacity, immunity while increasing susceptibility to diseases (6–8). Henceforth, appropriate nutritional strategies must be implemented to improve the reproductive potential and health of sows while maximizing breast milk quality.

Unfortunately, there is limited scientific research on the effects of Chinese herbal mixtures on perinatal sow reproductive performance, immunity, and breast milk quality. Bazhen powder (BZP), a traditional nourishing formula containing *Codonopsis pilosula* (Franch.) Nannf., *Atractylodes macrocephala* Koidz., *Poria cocos* (Schw.) Wolf, *Angelica sinensis* (Oliv.) Diels, *Ligusticum chuanxiong* Hort, *Paeonia lactiflora* Pall., *Rehmannia glutinosa* (Gaertn.) DC., and *Glycyrrhiza uralensis* Fisch., is currently used to treat diseases with deficiency of qi and blood (9). Qi plays a crucial role in regulating vital physiological processes and immune defense in the human body (9). Previous studies have shown that BZP can enhance performance and/or health in chicken (10), sows (11, 12), and other animals (13, 14). Qi-Zhu-Gui-Shao soothing liver and replenishing blood powder (QZGSP) is a self-prepared formula based on the syndrome of “*qi*-blood deficiency and Gan-*qi* stagnancy.” Its main ingredients are *Astragalus membranaceus* (Fisch.) Bunge, *A. macrocephala* Koidz., *A. sinensis* (Oliv.) Diels, *P. lactiflora* Pall., *Bupleurum chinense* DC. *P. cocos* (Schw.) Wolf. A previous study found that *A. membranaceus* is commonly utilized in the enhancement and treatment of various aliments as a medical agent and dietary supplement to fortify the spleen and restore vital energy (15). The primary natural active ingredient in *A. membranaceus* is Astragalus polysaccharide (APS), which exhibits a wide range of pharmacological properties such as immunostimulant, antioxidant, antibacterial, antiviral, and so on (16). *A. macrocephala* is known for its medicinal and edible values that can enhance spleen function, boost energy, eliminate dampness, promote urination, and prevent miscarriage (17). It contains polysaccharide as one of its active ingredients offering various of pharmacological benefits like immunoregulation, growth promotion, antioxidant effects, liver protection, and anti-tumor properties (18). *A. sinensis* is a traditional medicine and edible plant known for its efficacy in tonifying, nourishing, and activating blood (19). *A. sinensis* polysaccharide (ASP), identified as a crucial bioactive component of *A. sinensis* has been extensively investigated and found to possess significant pharmacological activities including immunomodulation, hepatoprotection, and antioxidant properties, among others (20). Paeoniae Radix Alba (PRA), known as Baishao in China, refers to the dried root of *P. lactiflora* Pall (PLP, also known as shaoyao). It has traditional pharmacological functions such as nourishing blood, attenuating liver diseases, regulating menstruation, and relieving pain (21). Modern pharmacological studies have revealed that PRA exhibits various functions, including anti-inflammation properties, liver protection, immunoregulation, and regulation of gut microbiota (22, 23). According to the theory of traditional Chinese medicine, Chaihu has the functions of soothing the liver and regulating qi, reducing water and dampness, relieving heat and pain, and supplementing Yang *qi* (24). It is commonly used to treat depression by “soothing the liver and alleviating melancholia” (25). Modern pharmacological studies have shown that Saikosaponins (SSs), as the main bioactive compounds in RB (*Radix Bupleuri*), possess various effects such as anti-inflammatory, anti-tumor, antioxidant, anti-viral, and hepatoprotective effects, etc. (26). Poria cocos refers to a functional edible and medicinal fungus, with Poria cocos polysaccharide (PCP) being one of its main active components. PCP and its derivatives exhibit various biological functions such as immunoregulatory, antioxidant, and hepatoprotective, etc. (27). In general, Chinese herbal mixtures contain multiple bioactive ingredients that synergistically enhance efficacy compared to using a single herb alone (28). However, the factors influencing the health status of sows before and after farrowing are exceedingly intricate; therefore, the efficacy of a singular Chinese herbal medicine in addressing these complex issues arising from sows before and after postpartum is often limited (29). Based on the multiple efficacies of the drugs contained in QZGSP, we hypothesized that QZGSP may have positive effects on reproductive performance, immunity and breast milk quality in perinatal sows. In addition, considering the biology, genetics, and dietary similarities shared by pigs and humans, this research may provide valuable scientific insights into the nutritional management of perinatal mothers.

## 2 Materials and methods

The experimental procedures of this study were approved by the Animal Care and Use Committee of Hebei Agricultural University (grant No. 2022161). The animal experiments were conducted at Weijia great grandparent farm located in Pingu district, Beijing, China.

### 2.1 Chinese herbal medicine mixture formula

The formula of Bazhen powder (BZP) used in this study consisted of *C. pilosula* (Franch.) Nannf, *A. macrocephala* Koidz, *P. cocos* (Schw.) Wolf, *A. sinensis* (Oliv.) Diels, *L. chuanxiong* Hort, *P. lactiflora* Pall, *R. glutinosa* (Gaertn.) DC, and *G. uralensis* Fisch, in a ratio of 1:1:1: 1:1:1: 1:1. The formula of Qi-Zhu-Gui-Shao soothing liver and replenishing blood powder (QZGSP) used in this study consisted of *A. membranaceus* (Fisch.) Bunge, *A. macrocephala* Koidz, *A. sinensis* (Oliv.) Diels, *P. lactiflora* Pall, *B. chinense* DC, and *P. cocos* (Schw.) Wolf, in a ratio of 6:3:3: 4:2:2. The aforementioned Chinese herbal mixture was commissioned for processing and manufacturing by Wuhan HVSEN Biotechnology Co., Ltd.

### 2.2 Animals, feeding and management

Sixty healthy pregnant sows (Large white) with fourth parity, similar backfat thickness, and expected confinement period were selected. They were then randomly divided into five groups (*n* = 12 per group): the control group received a basal diet, the TRT1 group received a basal diet supplemented with 2 kg/t BZP, the TRT2 group, TRT3 group, and the TRT4 group were each given the basic diet supplemented with 1 kg/t, 2 kg/t, and 3 kg/t of QZGSP, respectively. The trial lasted for 5 weeks, starting from the 100th day of pregnancy and continuing until the 21st day after delivery. The basic diets were prepared to fulfill the nutrient needs of sows as outlined by the NRC (2012) guidelines. The composition and nutrition level of these diets were presented in Table 1.

**Table 1:** The composition and nutrition level of the basal diets (air-dry basis, %).

Items	Late gestation	Lactation
Composition		
Corn	50.04	47.65
Barley	17.4	18
Soybean meal	17.2	19
Expanded soybean	6	6
Fish meal	2	2
NaCl	0.4	0.4
CaHPO_4_	1.4	1.4
Limestone	1.6	1.6
Lys	0.26	0.25
Soybean oil	2.7	2.7
Premix^1^	1	1
Total	100.00	100.00
Nutrition level^2^		
DM (MJ/kg)	14.20	14.30
CP	15.40	15.90
EE	5.00	5.10
Ash	5.80	5.90
*CF*	3.90	3.50
Ca	1.07	1.20
P	0.50	0.59
AP	0.40	0.45
Lys	1.14	1.17
Met	1.10	0.99

1 Premix during pregnancy: Cu 5 mg, I 0.15 mg, Fe 83 mg, Mn 20 mg, Zn 128 mg, VA 13,400 IU, VD_3_ 2,800 IU, Choline chloride 1,000 mg, VE 22.4 mg, VK_3_ 3 mg. Lactation premix: Cu 15 mg, Fe 82 mg, I 0.13 mg, Mn 20 mg, Zn 128 mg, VA 10,000 IU, VD_3_ 2,000 IU, VK_3_ 1.5 mg, VE 30 mg.

2 The DE was calculated and others were measured.

During the period from day 100 to day 110 of gestation, pregnant sows were allocated to individual stalls and provided with a controlled diet of 3 kg per day. On day 111th day of gestation, sows were shifted to individual farrowing pens equipped with an enclosed heated creep area. Starting from this day until delivery, each sow was given a daily feed allowance of 2.5 kg. The sows were not fed on the day farrowing. On the first day after delivery, sows were fed a lactation meal twice a day (at 7:30 AM and 2:30 PM), starting with an intake of 2.5 kg/d and then gradually increasing by 0.5 kg/d until *ad libitum* feeding was reached, respectively. The amount of feed consumed by each sow on a daily basis was measured during lactation in order to determine the their average daily feed intake (ADFI). Throughout the experiment, water was available *ad libitum* for all sows and suckling piglets. All sows were exposed to the same controlled growing environment where relative humidity and temperature are automatically regulated.

### 2.3 Data and sample collection

The number of piglets per litter, including total piglets, live piglets, healthy piglets, weak piglets (birth weight: <800 g), and stillborn fetuses, was recorded at birth. The amount of feed consumed by sows was monitored daily from day 1 to day 21 of lactation. Farrowing duration and interval were also recorded for each sow. The backfat thickness of each sow was measured on day 100 of gestation and again on days 0 and 21 of lactation using a digital backfat meter (Renco Lean-Meatier®, Renco Corporation, Minneapolis, MN, USA). The measurement point was located at a distance of 6.5 cm from the dorsal midline along the external tangent line of the last rib.

On days 0 and 21 of lactation, six sows were randomly chosen from each group. A total of 10 mL blood samples were taken from the marginal veins in the ear of each sow. These samples were subsequently divided into two parts: 5 mL of blood samples were placed in EDTA-K_2_ evacuated tubes, thoroughly mixed, and stored in a refrigerator at 4°C for future analysis, while the remaining 5 mL was transferred into sterile vacuum tubes. After being kept at room temperature for a duration of 30 min, the tubes were subjected to centrifugation at 3,000 rpm for 10 min at 4°C to obtain serum. The isolated serum was refrigerated at −20°C for subsequent examination.

On days 0, 11, and 21 of lactation, six sows were randomly selected from each group. Colostrum was obtained through manual expression from functional glands within 3 h after the start of farrowing. Milk collection followed an intramuscular injection of 20 IU oxytocin behind the ear. Approximately 20 mL of colostrum and milk samples were obtained per sow at each time point. Subsequently, the collected colostrum and milk samples were separated into two groups: 5 mL of milk samples were stored at −80°C for further analysis, while the remaining 15 mL of milk samples were centrifuged at 3,000 rpm for 20 min at a temperature of 4°C to obtain supernatant. The supernatant was refrigerated at −20°C for later analysis.

### 2.4 Determination of physiological and biochemical parameter of sows

The levels of white blood cells (WBC), red blood cells (RBC), and hemoglobin (HGB) in whole blood samples from sows in each group were measured using a veterinary blood cell analyzer (BC-5000, Shenzhen Mindray Biomedical Electronics Co., LTD, Shenzhen, China).

Serum concentrations of PROG (P), Estradiol (E2), and Prolactin (PRL) were determined through the double-antibody one-step sandwich assay utilizing porcine-specific ELISA kits (Shanghai Enzyme-linked Biotechnology Co., Ltd. Shanghai, China). The assays were performed according to the manufacturer’s instructions.

Serum levels of alanine aminotransferase (ALT) and aspartate aminotransferase (AST) were quantified using commercial kits (Nanjing Jiancheng Bioengineering Research Institute, Nanjing, China). All operations were carried out according to the manufacturer’s instructions.

### 2.5 Determination of serum, colostrum and milk immunity indexes of sows

The levels of cytokines (IL-4, IL-10, IL-1β, and TNF-α) and immunoglobulins (IgA, IgG) in the serum, colostrum, and milk of sows were determined using porcine-specific ELISA kits (Shanghai Enzyme-linked Biotechnology Co., Ltd. Shanghai, China), employing a double-antibody one-step sandwich assay. All operations were carried out according to the manufacturer’s instructions.

### 2.6 Determination of colostrum and milk composition of sows

Colostrum and milk samples from sows in each group were analyzed for the content of fat, protein, lactose and non-fat solids using a multifunctional dairy analyzer (MILKOSCAN FT1, FOSS Group, Denmark).

### 2.7 Sequencing of 16S ribosomal RNA amplified fragments from sow’s milk

Microbial genomic DNA was extracted from sow colostrum and milk using the Hipure Stool DNA Kit (Model D3141, Magen Biotechnology Co., Ltd., Guangzhou, China) according to the manufacturer’s instructions. The DNA concentration was measured using a Nanodrop 2000 spectrophotometer (Thermo Fisher Scientific Inc., DE, USA), and the integrity of the DNA was evaluated by agarose gel electrophoresis. Subsequently, PCR amplification of the V3-V4 variable region of the 16S rRNA gene was performed with primers (341F:5′-CCTACGGNGGCWGCAG-3′, 806R:5′-GGACTACHVGGTATCTAAT-3′) using isolated DNA as a template. Amplification products were visualized on 2% agarose gels and then purified using AMPure XP Beads (Beckman Coulter, Inc., USA) following the manufacturer’s instructions. Purified amplicons were mixed in equimolar amounts and subjected to paired-end sequencing on Novaseq 6000 platform following Gene Denovo Biotechnology Co., Ltd. (Guangzhou, China). Bioinformatic analysis was performed using the Omicsmart online platform.^1^

### 2.8 Statistics and analysis

Data from the experiments were organized and summarized using Excel software. Bar graphs were generated using GraphPad Prism 9.4.0 software (GraphPad Software Inc., San Diego, CA, USA), and the results were displayed as “mean ± standard deviation.” IBM SPSS 26.0 Software (SPSS Inc., Chicago, IL, USA) was used for one-way analysis of the data, and the experimental data were presented as “mean ± standard deviation,” with *p* < 0.05 indicating statistical significance.

## 3 Results

### 3.1 Effects of supplemental feeding of QZGSP to perinatal sows on reproductive performance in sows

As shown in Table 2, the number of piglets born alive and born healthy from sows in the TRT3 and TRT4 groups, as well as the litter weight at birth of piglets from the TRT3 group, exhibited significant increases compared to those in the CON group (*p* < 0.05). Sows in the TRT4, TRT3, and TRT1 groups demonstrated significantly lower farrowing duration and farrowing interval than those in the CON group (*p* < 0.05), while their average daily feed intake during lactation was significantly higher than that of the CON group (*p* < 0.05). Sows in the TRT4 and TRT3 groups also showed significantly lower farrowing duration and farrowing interval compared to those in the TRT1 group (*p* < 0.05), along with a significantly higher average daily intake during lactation than that of the TRT1 group (*p* < 0.05). Sows in the TRT1, TRT3, and TRT4 groups experienced a significant decrease in backfat thickness loss during lactation and a significant increase in average milk yield compared with sows in the CON group (*p* < 0.05). Sows in the TRT3 and TRT4 groups had a significant increase in average milk yield compared with sows in the TRT1 group (*p* < 0.05).

**Table 2:** Effects of QZGSP on sow’s reproductive performance during late gestation and lactation.

Items	CON	TRT1	TRT2	TRT3	TRT4	*p*-value
Litter, no						
Total born	14.42	14.75	14.58	14.91	15.08	0.612
Born alive	13.42	13.92	13.67	14.17^*^	14.42^*^	<0.01
Healthy born	12.92	13.42	13.17	13.58^*^	13.75^*^	<0.01
BW < 800 g	0.50	0.50	0.50	0.58	0.67	0.791
Still born	0.92	0.75	0.83	0.58	0.58	0.978
BW, kg						
Piglet birth weight	1.31	1.30	1.33	1.29	1.28	0.260
Litter birth weight	17.52	18.11	18.14	18.32	18.48^*^	0.161
Farrowing time, min						
Farrowing duration	266.33	238.25^*^	255.75^#^	205.42^*#^	214.83^*#^	*<*0.01
Farrowing interval	18.58	15.72^*^	17.56^#^	13.63^*#^	13.98^*#^	*<*0.01
**ADFI at lactation, kg**	5.52	5.77^*^	5.58^#^	5.97^*#^	5.89^*#^	*<*0.01
Backfat thickness, mm						
Day 100 of lactation	17.58	17.42	17.33	17.50	17.58	0.977
After farrowing	18.25	18.50	18.33	18.83	18.75	0.247
At weaning	14.58	15.75^*^	14.75^#^	16.42^*^	16.25^*^	<0.01
Lactation loss	3.67	2.75^*^	3.58^#^	2.33^*^	2.50^*^	<0.01
**Average milk yield, kg/d**	6.28	7.17^*^	6.31^#^	8.07^*#^	7.71^*#^	<0.01

BW, body weight; ADFI, average daily feed intake.

Control (CON, basal diet), treatment group 1 (TRT1, basal diet + 2 kg/t BZP), treatment group 2 (TRT2, basal diet + 1 kg/t QZGSP), treatment group 3 (TRT3, basal diet + 2 kg/t QZGSP), and treatment group 4 (TRT4, basal diet + 3 kg/t QZGSP).

“*”: indicates a significant difference compared with mean values in CON (*p* < 0.05), “^#^”: indicates a significant difference compared with mean values inTRT1 (*p* < 0.05).

### 3.2 Effects of supplemental feeding of QZGSP to perinatal sows on physiological and biochemical parameters in the blood or serum of sows

**Figure 1:**
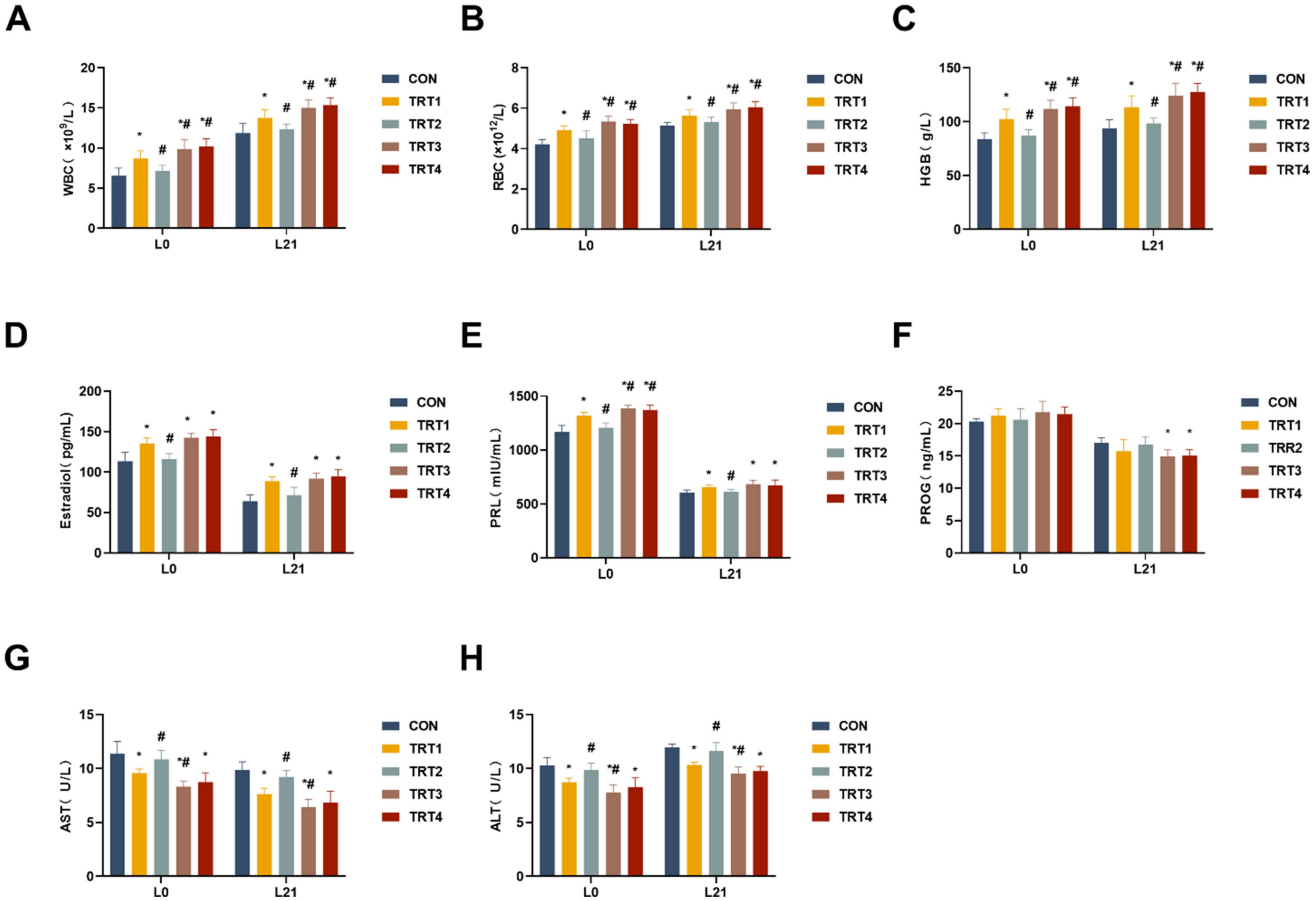
Effects of QZGSP on physiological and biochemical parameters in the blood or serum of sows during lactation. **(A–C)** Levels of WBC, RBC, and HGB in complete blood of sows on days 0 and 21 of lactation. **(D–F)** Levels of serum Estradiol, PRL, and PROG of sows on days 0 and 21 of lactation. **(G,H)** Levels of serum AST and ALT of sows on days 0 and 21 of lactation. CON, basic diet; TRT1 group, basic diet supplemented with 2 kg/t BZP; TRT2 group, basic diet supplemented with 1 kg/t QZGSP; TRT3 group, basic diet supplemented with 2 kg/t QZGSP; TRT4 group, basic diet supplemented with 3 kg/t QZGSP. “*”: indicates a significant difference compared with CON group (*p* < 0.05), “#”: indicates a significant difference compared with TRT1 group (*p* < 0.05). The same to below.

To assess the impacts of QZGSP on changes in complete blood or serum parameters of sows, levels of WBC, RBC, HGB, PRL, E2, AST, and ALT were measured. As shown in Figures 1A–C, the levels of WBC, RBC, and HGB in the complete blood of sows from the TRT4, TRT3, and TRT1 groups were significantly higher compared to the CON group on days of 0 and 21 lactation (*p* < 0.05). A significant increase was noted in total WBC count as well as RBC count and HGB level in the TRT4 and TRT3 groups compared to the TRT1 group (*p* < 0.05). The levels of E2 and PRL in the serum of sows from the TRT4, TRT3, and TRT1 groups were significantly higher than those in the CON group on days 0 and 21 of lactation (*p* < 0.05), as depicted in Figures 1D,E. On day 0 of lactation, the content of PRL in the serum of sows from the TRT4 and TRT3 groups were significantly greater than that in the TRT1 group (*p* < 0.05). On day 21 of lactation, as shown in Figure 1F, the level of PROG in the serum of sows from the TRT4 and TRT3 groups was significantly higher than that in the CON group (*p* < 0.05). The serum levels of AST and ALT activities in sows from the TRT4, TRT3 and TRT1 groups were significantly lower than those in the CON group on days 0 and 21 of lactation (*p* < 0.05), as shown in Figures 1G,H. In addition, AST and ALT activities were notably lower in the TRT3 group compared to the TRT1 group on days 0 and 21 of lactation (*p* < 0.05).

### 3.3 Effects of supplemental feeding of QZGSP to perinatal sows on serum immunity of sows

**Figure 2:**
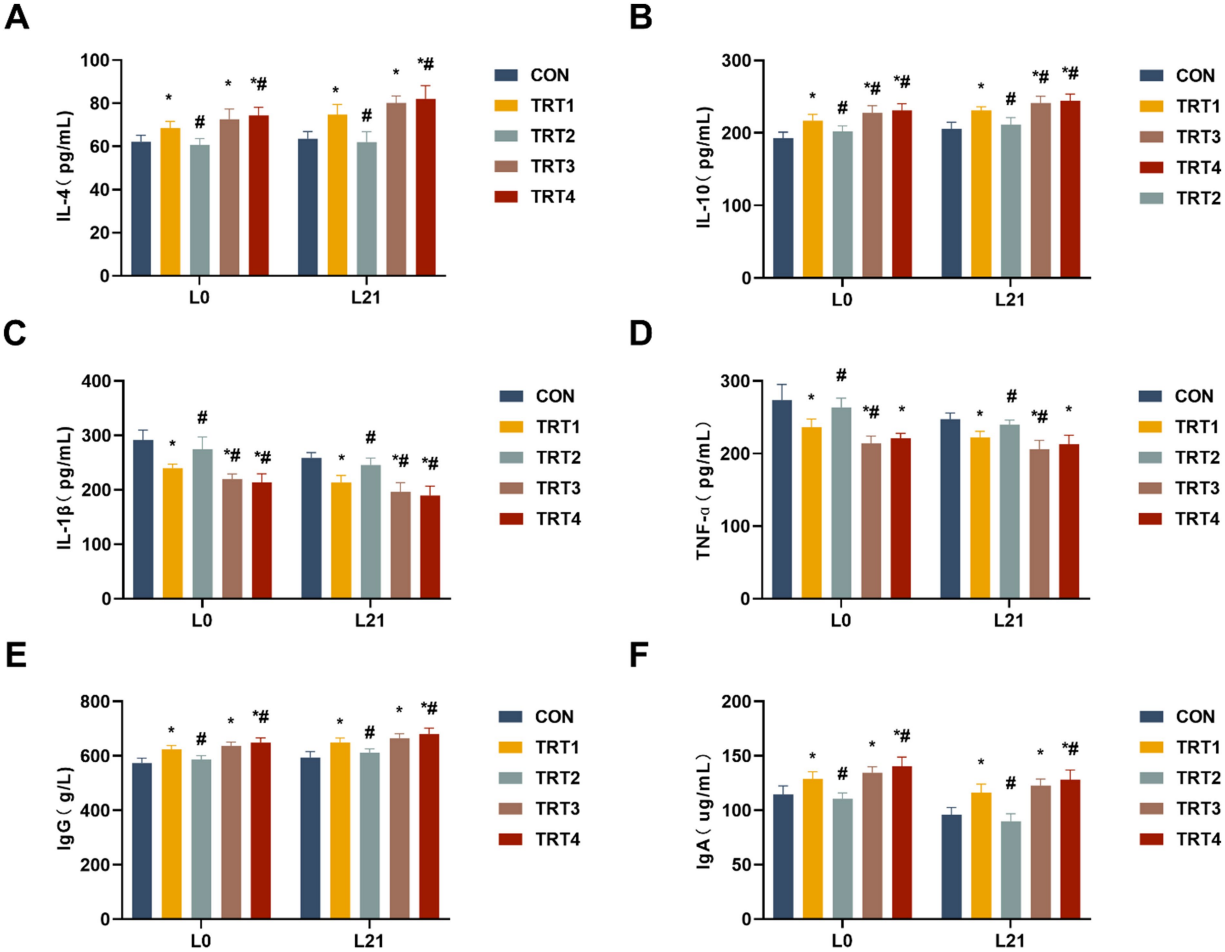
Effects of QZGSP on the immunity of sows. **(A–D)** Concentrations of IL-4, IL-10, IL-1β, and TNF-α in the serum of sows. **(E,F)** Concentrations of immunoglobulins IgG and IgA in serum of sows.

The impacts of QZGSP on the modulation of immunity were assessed by quantifying serum cytokines, including IL-4, IL-10, IL-1β, and TNF-α. As shown in Figures 2A,B, sows from the TRT4, TRT3, and TRT1 groups exhibited significantly higher serum concentrations of IL-4 and IL-10 compared to those in the CON group on days 0 and 21 of lactation (*p* < 0.05). Sows from the TRT4 group had significantly higher serum concentrations of IL-4 than those from the TRT1 group on days 0 and 21 of lactation (*p* < 0.05), while sows from the TRT4 and TRT3 groups had significantly higher serum concentrations of IL-10 than those from the TRT1 group on days 0 and 21 of lactation (*p* < 0.05).

The sows from the TRT4, TRT3, and TRT1 groups exhibited significantly lower serum levels of IL-1β and TNF-α compared to the CON group on days 0 and 21 of lactation (*p* < 0.05), as depicted in Figures 2C,D. The levels of IL-1β in the serum of sows from the TRT4 and TRT3 groups, as well as the levels of TNF-α in the serum of sows from the TRT3 group, were significantly lower than that in the TRT1 group on days 0 and 21of lactation (*p* < 0.05).

The concentrations of IgG and IgA in the serum of lactating sow were measured. As shown in Figures 2E,F, compared to sows in the CON group, sows in the TRT1, TRT3, and TRT4 groups exhibited significantly increased concentrations of IgG and IgA in their serum on days 0 and 21 of lactation (*p* < 0.05). Sows in the TRT4 group showed significantly higher concentrations of IgG and IgA in their serum on days 0 and 21 of lactation compared to those in the TRT1 group (*p* < 0.05).

### 3.4 Effects of supplemental feeding of QZGSP to perinatal sows on nutrient composition of colostrum and milk of sows

**Figure 3:**
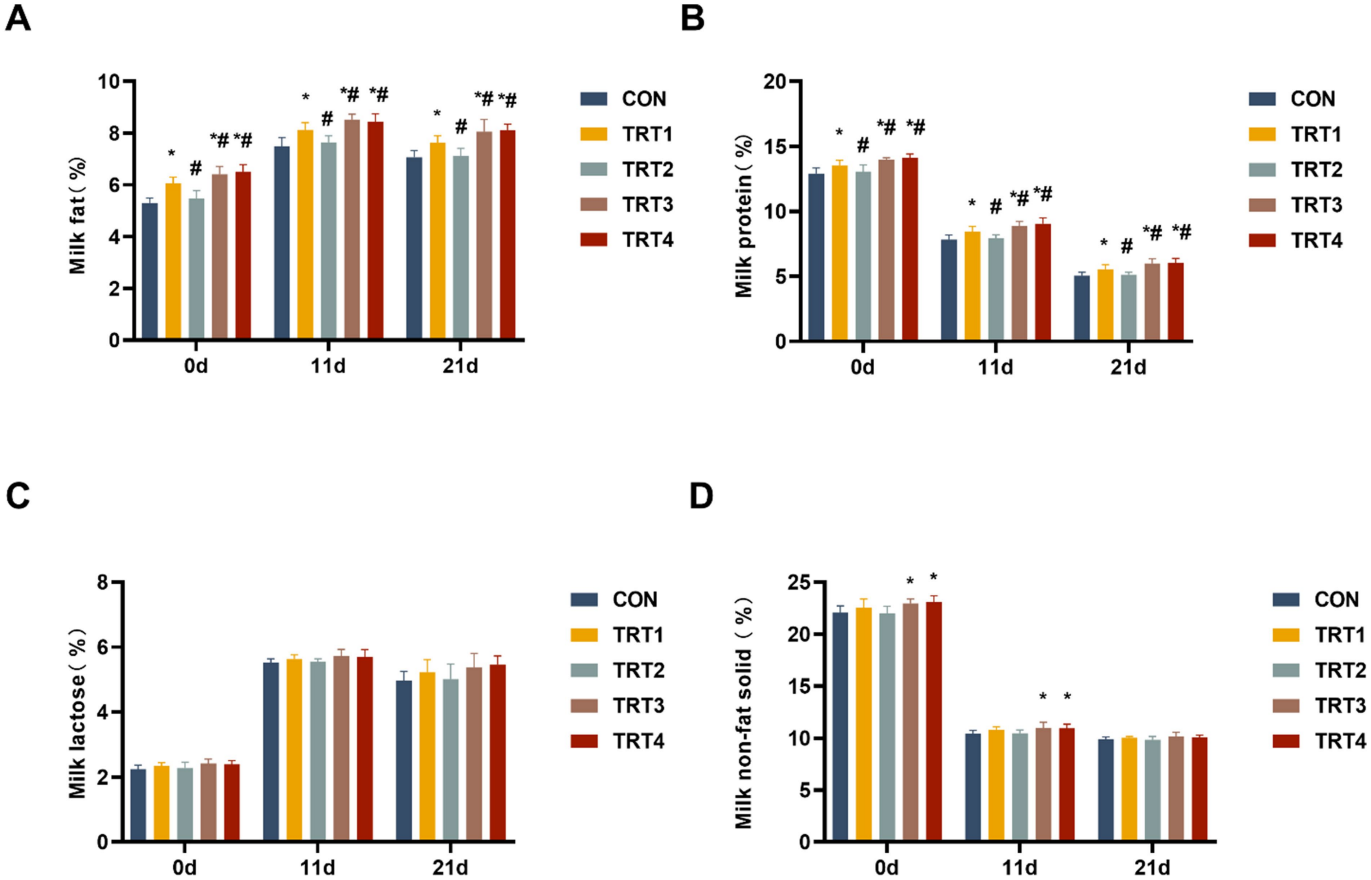
Effects of QZGSP on nutrient composition of colostrum and milk of sows. **(A)** Milk protein content. **(B)** Milk fat content. **(C)** Milk lactose content. **(D)** Milk non-fat-solid content.

The nutritional composition of colostrum and milk from sows was evaluated on days 0, 11, and 21 of lactation. As shown in Figures 3A,B, the concentration of fat and protein in sow colostrum and milk from TRT4, TRT3, and TRT1 groups was significantly higher than that in the CON group (*p* < 0.05). The concentration of fat and protein in sow colostrum and milk from TRT4 and TRT3 group was significantly higher than that in the TRT1 group (*p* < 0.05). Moreover, Figure 3C indicates that there were no significant differences in the concentrations of lactose in sow colostrum and milk among the different groups (*p* > 0.05). As shown in Figure 3D, the concentration of non-fat solid in sow colostrum and milk from TRT4 and TRT3 groups was significantly higher than that in the CON group on days 0 and 11 of lactation (*p* < 0.05).

### 3.5 Effects of supplemental feeding of QZGSP to perinatal sows on colostrum and milk immune indexes of sows

**Figure 4:**
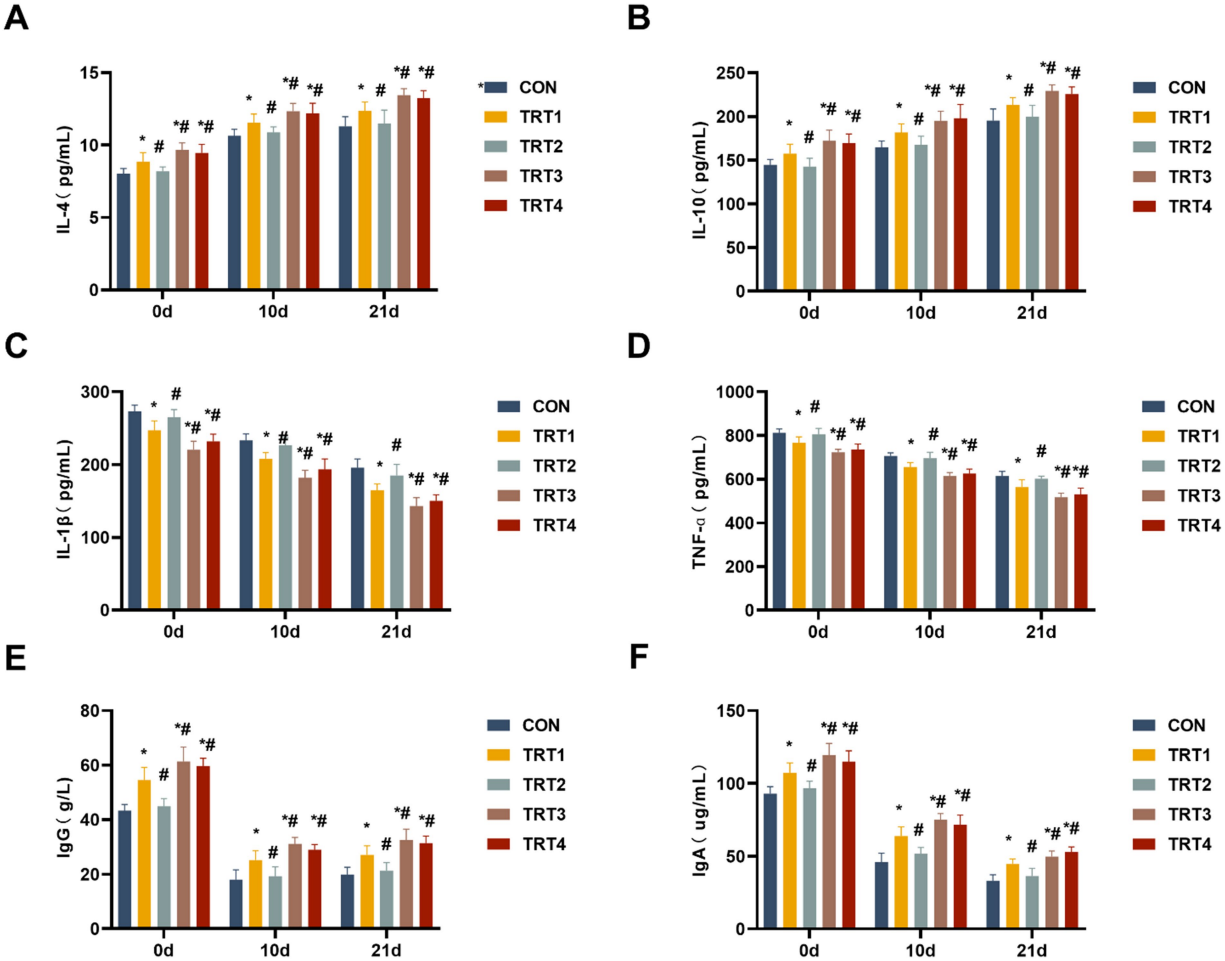
Effects of QZGSP on the colostrum and milk immune indexes of sows. **(A–D)** Concentrations of IL-4, IL-10, IL-1β, and TNF-α in the colostrum and milk of sows. **(E,F)** Concentrations of immunoglobulins IgG and IgA in the colostrum and milk of sows.

Concentrations of cytokines (IL-4, IL-10, IL-1β, and TNF-α) in colostrum and milk were determined to investigate the impacts of QZGSP on changes in immune indexes. As shown in Figures 4A–D, sows from the TRT4, TRT3, and TRT1 groups exhibited significant increases in the levels of IL-4 and IL-10 in colostrum and milk, as well as significant decreases in the levels of IL-1β and TNF-α in colostrum and milk compared to the CON group on days 0, 11, and 21 of lactation (*p* < 0.05). Sows from the TRT4 and TRT3 groups showed significantly higher levels of IL-4 and IL-10 in colostrum and milk while also displaying significantly lower levels of IL-1β, and TNF-α in colostrum and milk, compared to the TRT1 group on days 0, 11, and 21 of lactation (*p* < 0.05).

The concentrations of IgG and IgA in colostrum and milk from lactating sows were measured. As presented in the Figures 4E,F, the concentration of IgG and IgA in colostrum and milk from the TRT4, TRT3, and TRT1 groups was significantly higher than that in the CON group (*p* < 0.05). The concentrations of IgG and IgA in colostrum and milk from the TRT4 and TRT3 groups were significantly higher than those in the TRT1 group (*p* < 0.05).

### 3.6 Effects of supplemental feeding of QZGSP to perinatal sows on microbial flora composition in colostrum and milk of sows

**Figure 5:**
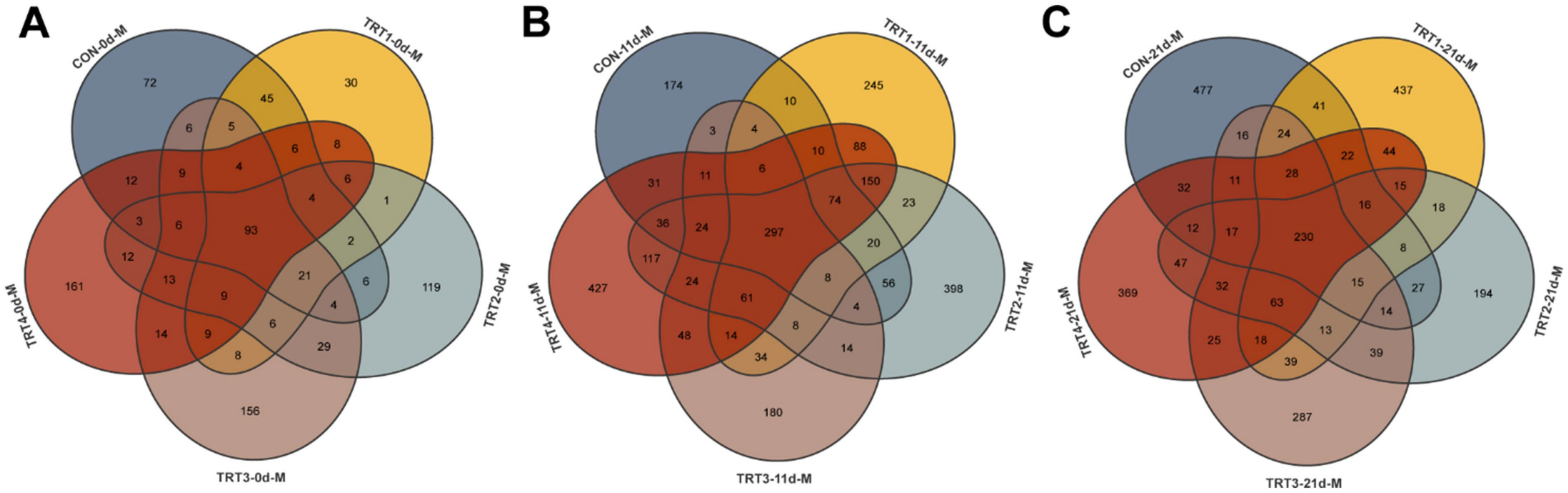
The Venn diagram for OTUs of the milk microbiota of sows among different groups. The Venn diagram for OTUs of milk microbiota of sows on days 0 **(A)**, 11 **(B)** and 21 **(C)** of lactation.

The effects of dietary QZGSP supplementation with QZGSP on the diversity and richness of microorganisms in sow colostrum and milk were examined. We collected 298, 257, 334, 392 and 369 OUT samples from the CON, TRT1, TRT2, TRT3, and TRT4 groups, respectively. Among them, there were 93 OUT samples shared by all five groups (Figure 5A). We also collected 768, 1,052, 1,314, 740, and 1,418 OTU samples from the CON, TRT1, TRT2, TRT3, and TRT4 groups, with 297 OUT samples shared among the five groups (Figure 5B). We obtained 990, 1,031, 760, 871, and 981 OTU samples from the CON, TRT1, TRT2, TRT3, and TRT4 groups, respectively. Among them, there were 230 OUT samples shared by all five groups (Figure 5C).

The alpha diversity indices of colostrum and milk microbiota for sows are presented in Table 3. On day 11 of lactation, the Chao1 index and Ace index were significantly higher in the TRT3 and TRT4 groups compared to the CON group (*p* < 0.05), while the Simpson index was notably lower in the TRT3 group than in the CON group (*p* < 0.05). On day 21 of lactation, both Chao1 and Ace indices were significantly higher in the TRT2, TRT3, and TRT4 groups compared with those in the TRT1 group (*p* < 0.05). However, Shannon index was significantly lower in the TRT2 group than that of both CON and TRT1 groups (*p* < 0.05), whereas Simpson index was also significantly lower only in the TRT2 group than that of the TRT1 group (*p* < 0.05).

**Table 3:** Effects of dietary QZGSP supplementation on alpha diversity of milk microbiota for sows.

Items		CON	TRT1	TRT2	TRT3	TRT4	*p*-value
0d	Chao1	519.47	485.80	414.39	424.18	472.38	0.253
	Ace	540.32	514.63	442.21	447.25	493.80	0.178
	Shannon	3.18	2.67	2.87	3.02	3.13	0.324
	Simpson	0.82	0.72	0.74	0.76	0.79	0.350
11d	Chao1	777.91	1,040.89	1,042.48	688.99	1,241.56^*^	0.101
	Ace	822.00	1,107.10	1,082.71	731.89	1,290.63^*^	0.104
	Shannon	6.75	6.51	6.69	5.38	7.18	0.133
	Simpson	0.98	0.96	0.96	0.91^*^	0.98	0.160
21d	Chao1	937.99	635.84	923.45^#^	1,021.50^#^	993.69^#^	0.009
	Ace	980.72	678.18	990.76^#^	1,084.43^#^	1,075.49^#^	0.007
	Shannon	5.19	6.05	3.60^*#^	5.44	5.30	0.039
	Simpson	0.86	0.94	0.71^#^	0.90	0.91	0.037

Control (CON, basal diet), treatment group 1 (TRT1, basal diet + 2 kg/t BZP), treatment group 2 (TRT2, basal diet + 1 kg/t QZGSP), treatment group 3 (TRT3, basal diet + 2 kg/t QZGSP), and treatment group 4 (TRT4, basal diet + 3 kg/t QZGSP).

“*”: indicates a significant difference compared with mean values in CON (*p* < 0.05), “^#^”: indicates a significant difference compared with mean values inTRT1 (*p* < 0.05).

**Figure 6:**
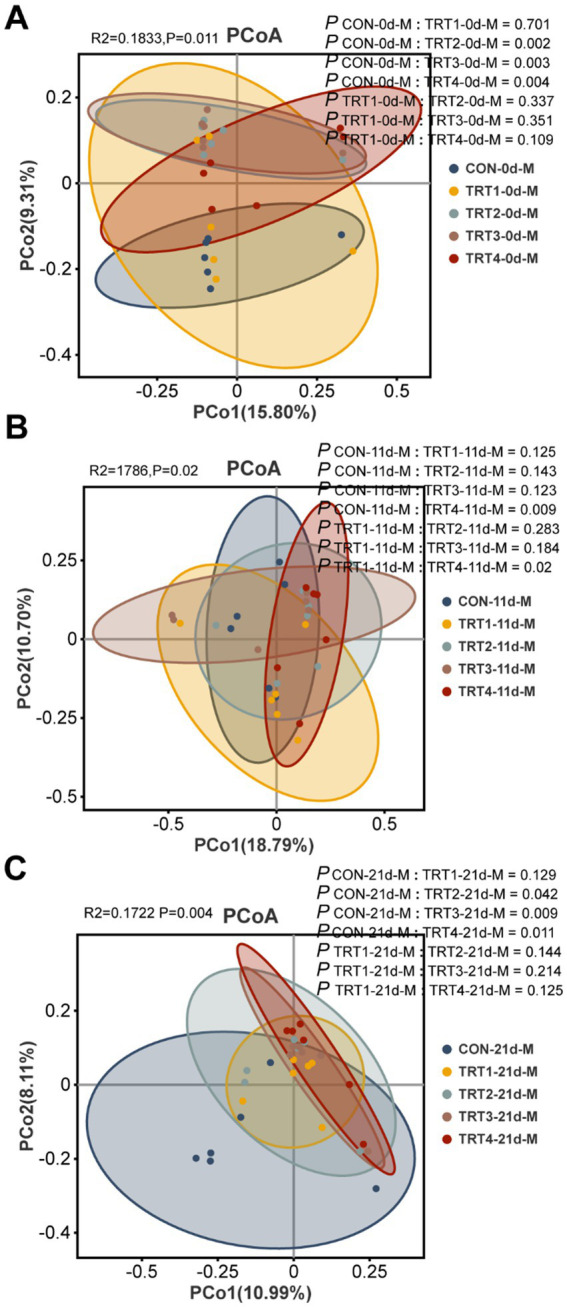
Effects of dietary QZGSP supplementation on beta-diversity of milk microbiota for sows. PCoA for sow among different groups based on unweighted_unifrac distance analysis on days 0 **(A)**, 11 **(B)** and 21 **(C)** of lactation. Inter-group β-diversity comparisons using Adonis.

The effects of QZGSP on beta diversity of milk microbiota were explored using principal coordinate analysis (PCoA) to assess group differences. PCoA was performed based on the unweighted_unifrac distance of OUT relative abundance in sow milk microbiota. As depicted in Figure 6A, there was a distinct separation between the CON and TRT2 groups (Adonis: *p* = 0.002), as well as between the CON and TRT3 groups (Adonis: *p* = 0.017), and the CON and TRT4 groups (Adonis: *p* = 0.004). Similarly, in Figure 6B, a distinct separation was observed between the CON and TRT4 groups (Adonis, *p* = 0.009), along with the separation between the TRT1 and TRT4 groups (Adonis, *p* = 0.02). Furthermore, Figure 6C revealed separations between the CON and TRT2 groups (Adonis: *p* = 0.042), the CON and TRT3 groups (Adonis: *p* = 0.009), as well as the CON and TRT4 groups (Adonis: *p* = 0.011).

### 3.7 Effects of supplemental feeding of dietary QZGSP supplementation on the microbial flora structure in colostrum and milk of sows

**Figure 7:**
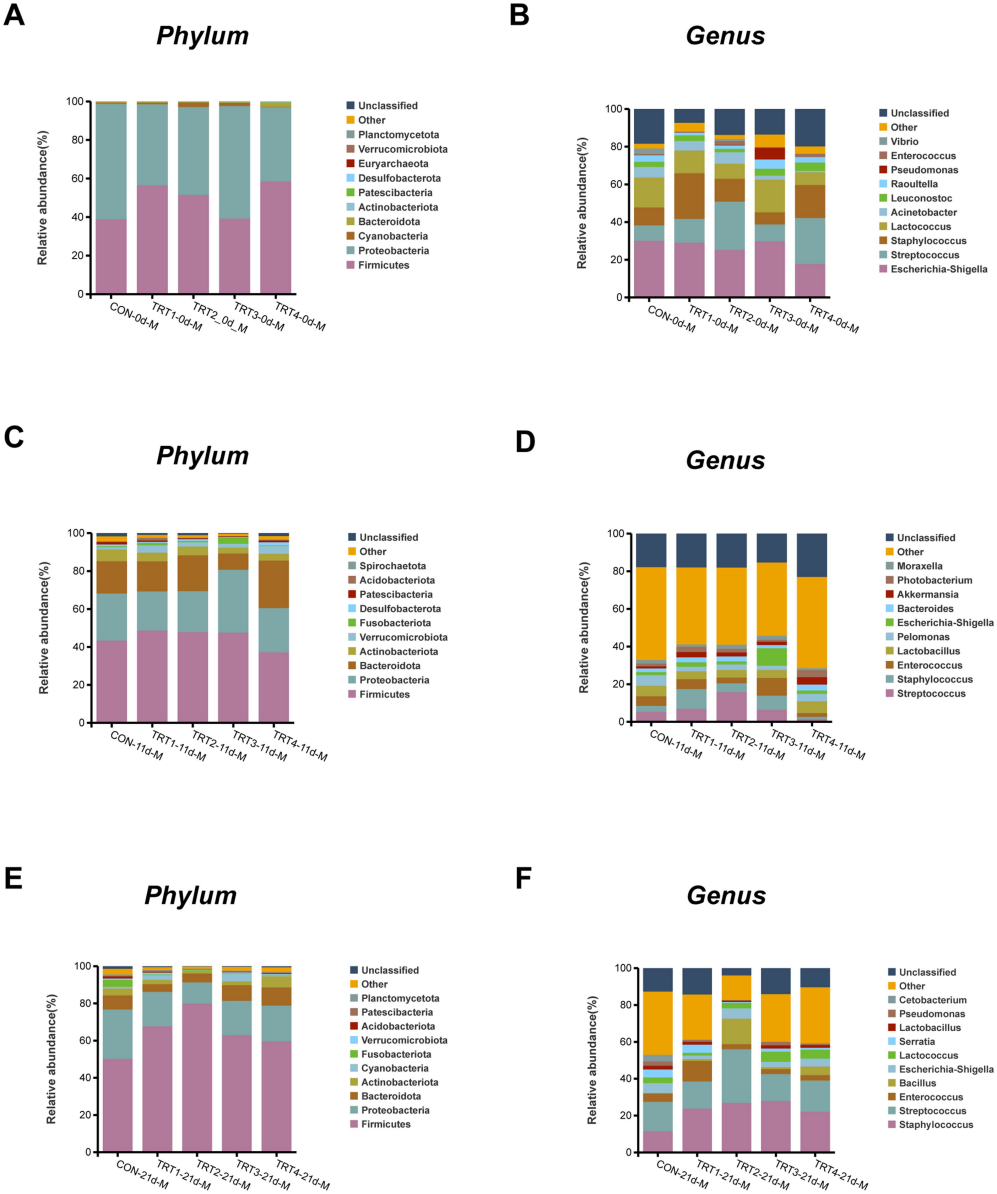
Effects of dietary QZGSP supplementation on the composition of milk microbiota in sows among different groups. The phylum-level composition of average relative abundance milk microbiota on day 0 **(A)**, 11 **(C)** and 21 **(E)** of lactation. The genus-level composition of average relative abundance milk microbiota on days 0 **(B)**, 11 **(D)** and 21 **(F)** of lactation. Values are expressed as mean ± SD, *n* = 6.

The composition of microflora species in sow milk from different groups at the phylum and genus levels were illustrated in Figure 7. As shown in Figures 7A,C,E, the dominant phylum observed throughout lactation were *Firmicutes*, *Proteobacteria*, *Bacteroidetes*, and *Actinobacteria*. There was an increase in the abundance of *Firmicutes* in the TRT3 group on day 21 of lactation compared to days 0 and 11. As depicted in Figures 7B,D,F, *Escherichia-Shigella*, *Streptococcus*, and *Staphylococcus* emerged as the predominant genera within each group during lactation. On day 0 of lactation, the TRT4 group had lower levels of Escherichia-Shigella than the other groups. In addition, the TRT3 group had lower levels of *Staphylococcus* and *Streptococcus* on day 0 lactation compared to the other groups.

**Figure 8:**
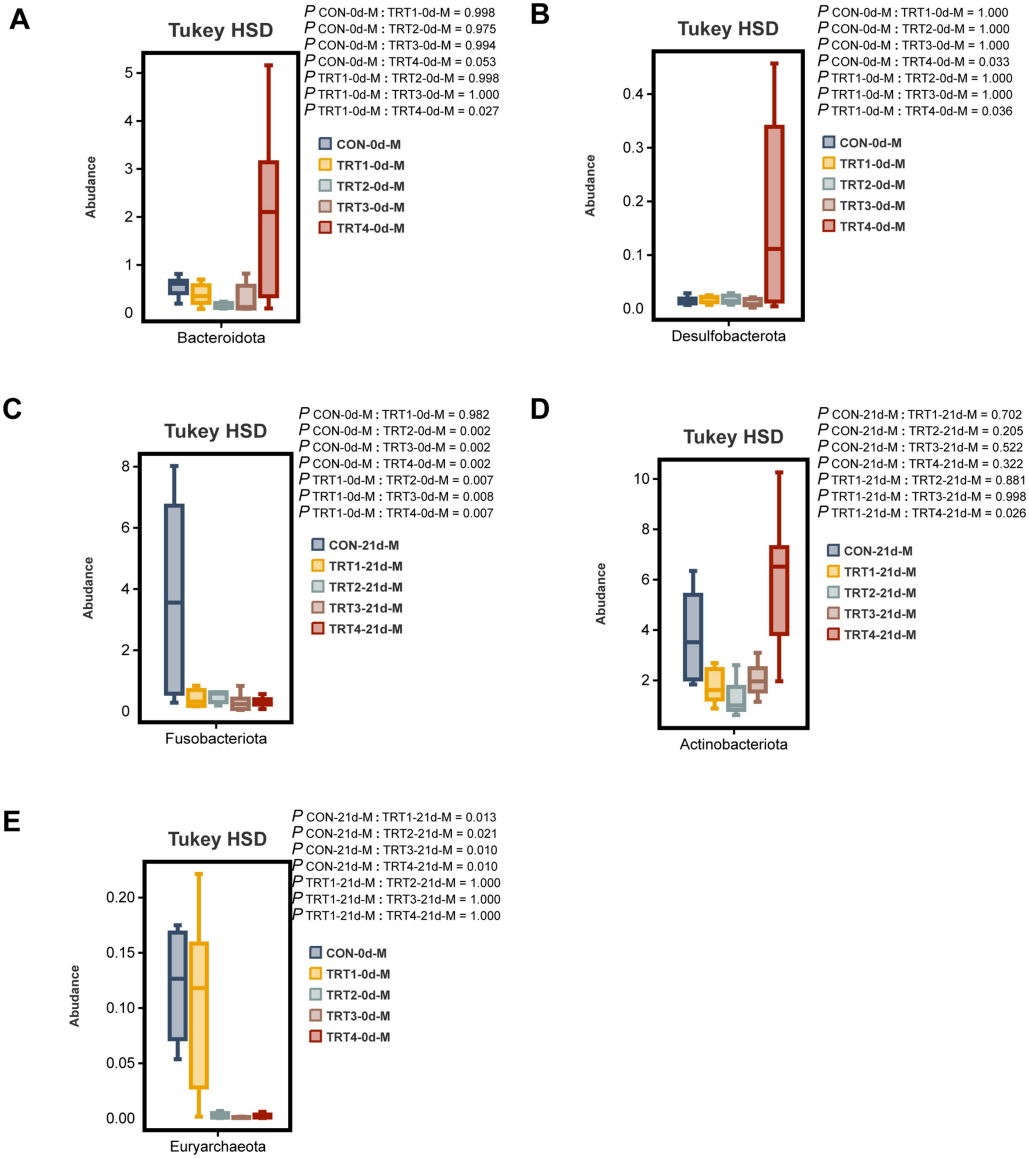
Effects of dietary supplementation with QZGSP on differences in milk microbiota of sows at the phylum level among different groups. The significant changes in the abundance of bacterial phyla found in milk **(A–E)**. Values are expressed as mean ± SD, *n* = 6.

The comparative analysis was conducted among different groups to examine the top 10 dominant bacteria at the phylum level in sow colostrum and milk. On day 0 of lactation, the TRT4 group exhibited a significantly higher relative abundance of *Bacteroidota* compared to the TRT1 group (*p* < 0.05, Figure 8A). On day 0 of lactation, the TRT4 group showed a significantly higher relative abundance of *Desulfobacterota* compared to both CON and TRT1 groups (*p* < 0.05, Figure 8B), while the relative abundance of *Euryarchaeota* in TRT2, TRT3, and TRT4 groups was significantly lower than that in CON and TRT1 groups (*p* < 0.05, Figure 8C). On day 21 of lactation, the relative abundance of *Actinobacteriota* in the TRT4 group was significantly higher than that in the TRT1 group (*p* < 0.05, Figure 8D), whereas *Fusobacteriota* had a significantly higher relative abundance in the CON group on day 21 of lactation compared to other groups (*p* < 0.05, Figure 8E).

**Figure 9:**
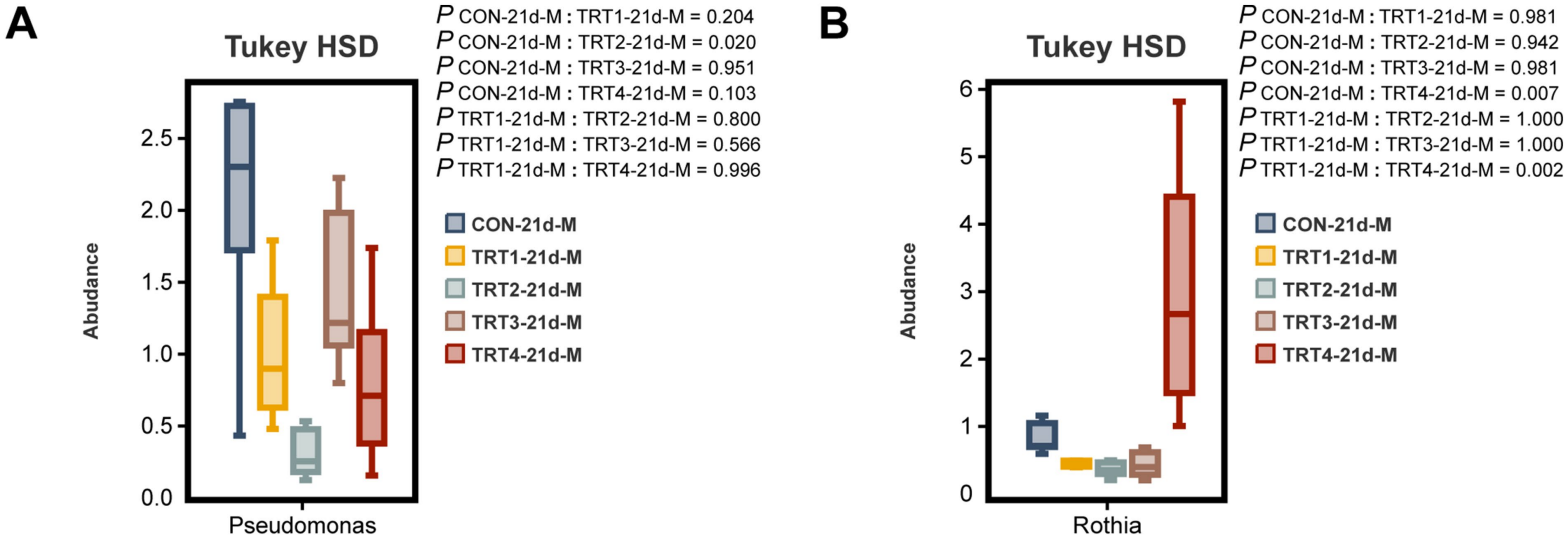
Effects of dietary supplementation with QZGSP on differences in milk microbiota of sows at the genus level among different groups. The significant changes in the abundance of bacterial genera found in milk **(A,B)**. Values are expressed as mean ± SD, *n* = 6.

The comparative analysis was conducted among different groups to examine the top 10 dominant bacteria at the genus level in sow colostrum and milk. As depicted in Figure 9A, there was a significant decrease in the relative abundance of *Pseudomonas* in the TRT2 group compared to that in the CON group on day 21 of lactation (*p* < 0.05). Moreover, as shown in Figure 9B, the relative abundance of *Rothia* on day 21 of lactation was significantly higher in the TRT4 group than the CON and TRT1 groups (*p* < 0.05).

## 4 Discussion

The health status of sows is crucial for modern intensive pig farming enterprises. The reproductive performance of sows serves as a vital indicator of the economic efficiency of pig enterprises. Chinese herbal medicines contain a wide range of bioactive compounds that offer various nutritional and health benefits to animals, making them commonly utilized as feed additives in livestock (30). Prior researches have shown that the utilization of CHMs as potential alternatives to antibiotic growth promoters (AGPs) for enhancing the reproductive performance of pigs has gained widespread recognition (31). In this study, we found that supplementing perinatal sows with 2 kg/t and 3 kg/t QZGSP significantly improved their reproductive performance, immunity, and breast milk quality. Overall, QZGSP is a beneficial feed additive for perinatal sows.

According to the theory of traditional Chinese veterinary medicine, “*qi* and blood deficiency stagnation” is a major issue for sows before and after farrowing, which directly affects the health status and reproductive performance of sows. Therefore, the focus of sow healthcare before and after farrowing should be on addressing “*qi* and blood” through the use of Chinese herbs that replenish qi and blood, soothe the liver, strengthen the spleen, ultimately leading to improve production performance and overall health of sows. Some experiments have demonstrated that the including *A. membranaceus* in the basal diet leads to an augmentation in animal feed intake (32), enhances animal body condition, and promotes weight gain (33). Supplementing postpartum dairy cows with *A. membranaceus* significantly alleviated the extent of weight loss (34). Wu et al. (35) reported that a combination of soybean isoflavone (SI) and astragalus polysaccharide (APS) significantly improved average daily feed intake and lactation yield in lactating sows. *A. macrocephala Koidz* improved average daily feed intake in piglets (36). Dietary supplementation with 3% Aerial parts of *A. sinensis* significantly increased feed consumption in broiler (37). Dietary supplementation with peony pollen improved feed intake in common carp (38). Dietary supplementation of *A. membranaceus or/and B. chinense* significantly increased the specific growth rate and feed conversion ratio of shrimp (39). Dietary supplementation of *Bupleurum falcatum L saikosaponins* restored the growth performance of chickens exposed to NH_3_ through enhancing DWG and reducing FCR (40). The feed intake and body condition of periparturient sows are crucial for the lactation performance of sows and the growth and development of piglets (41). Sow farrowing duration is correlated with hemoglobin concentration (42), while highly productive sows may experience decrease hemoglobin levels after farrowing, resulting in weakness, fatigue, loss of appetite, prolonged farrowing time, and increased stillbirth rates (43). In our study, we found that supplemental feeding of QZGSP significantly increased hemoglobin concentration in whole blood of sows. In this study, we found that supplemental feeding of 2 kg/t and 3 kg/t QZGSP to perinatal sows significantly improved average daily feed intake and milk yield in sows while reduced farrowing duration in sows and backfat thickness loss. This phenomenon may be attributed to the synergistic effect of multiple bioactive ingredients in Chinese herbs of QZGSP, which increased feed intake, lactation performance and hemoglobin concentration of sows, thus shortening farrowing and reducing backfat thickness loss, and thus improving reproductive performance of sows to a certain extent.

Each component in the blood serves as the material basis for metabolism, and changes in its content can reflect alterations in metabolic function, nutrient metabolism, as well as the functioning of tissues and organs within the body. Ultimately, these changes can indicate the health or disease status of livestock and poultry (44). Aberrant levels of white blood cells (WBC) may suggest potential immune dysfunction within the body, thus signifying their significance as immunological markers (45). Red blood cells (RBC) play essential roles in transporting oxygen, carbon dioxide, and modulating the immune system. Hemoglobin (HGB) is crucial for their transport function. Lan et al. (46) reported that the use of an herbal mixture containing *A. membranaceus*, *Codonopsis pilosula*, and *allicin* as a dietary supplement significantly increased white blood cell concentrations in finishing pigs compared to the control group. Jiang et al. (47) reported that APS significantly enhances the immunological function of erythrocytes in chicken infected with infectious Bursa disease virus (BDV). *Atractylodes lancea rhizome polysaccharide* (ALP) effectively increased the blood cell count, including white blood cells, hemoglobin, and red blood cells, in immunosuppressed mice (48). Angelica sinensis at doses of 200 and 400 mg/kg/d significantly increased the levels of white blood cells (WBC) and red blood cells (RBC) in mice with aplastic anemia (49). In this study, supplemental feeding of 2 kg/t and 3 kg/t QZGSP to perinatal sows significantly elevate WBC, RBC, and HGB concentrations within the normal physiological range. These findings are consistent with prior research reports and suggest that supplemental feeding of QZGSP to perinatal sows enhances the body’s ability to transport oxygen, and a large number of red blood cells and hemoglobin combine to transport to various tissues and organs, thereby improving the body’s metabolic capacity as well as having a significant effect on the body’s immune performance.

The synthesis and secretion of milk are complex physiological processes regulated by the neuroendocrine system and involve a variety of endocrine hormones (50). After delivery, the concentration of estrogen and progesterone in the blood is greatly reduced in sows. This reduction lifts their inhibitory effect on prolactin, allowing prolactin to bing to mammary follicular epithelial receptors, thereby initiating and maintaining lactation. In addition, the sow serum prolactin concentrations increase due to the stimulation caused by piglets suckling on the sows. Previous studies have reported serum prolactin levels in primiparous sows are positively correlated with colostrum production, suggesting that higher concentrations of prolactin lead to an increase in colostrum production (51, 52). Other studies have shown that supplemental feeding of Astragalus to dairy cows after parturition can increase the level of prolactin secretion and milk production in dairy cows (34). Flavonoids found in Astragalus have estrogen-like effects (53, 54), which can stimulate prolactin synthesis and secretion in lactating sows by affecting the gonadal axis (55). In the study, supplemental feeding of 2 kg/t and 3 kg/t QZGSP to periparturient sows significantly increased estrogen and prolactin levels to some extent, which may be related to the regulation of hormone metabolism levels in sows after absorbing different levels of active components of the herbs in QZGSP. The levels of various hormones are different in different production stages of sows, and the specific mechanism of QZGSP needs to be further investigated.

AST and ALT are commonly chosen as key indicators for diagnosing liver diseases in clinical practice (56). Elevation of plasma AST and ALT generally reflects hepatocyte damage (57). Previous research has demonstrated that Angelica and *A.* Polysaccharide can dramatically lower serum AST and ALT levels in CCl_4_-treated mice, consequently reducing CCl_4_-induced liver injury (58). *A. radix* could decrease serum AST and ALT levels as well as ameliorate hepatic pathological damages caused by cisplatin (59). Adding 200 mg/kg or 400 mg/kg prepared *A. macrocephala* Koidz to a high-energy and low-protein laying hens diet reduces the levels of AST and ALT in plasma (60). *R.* Bupleuri exerts a significant hepatoprotective effect against acetaminophen (APAP)-induced acute liver injury by reducing the levels of AST and ALT in serum (61). Experiments on ethanol-induced acute liver injury showed that Angelica sinensis polysaccharide (ASP) reduced the expression of aspartate aminotransferase (AST) and alanine aminotransferase (ALT) (62). In this study, supplemental feeding of 2 kg/t and 3 kg/t QZGSP significantly decreased serum AST and ALT levels in sows, which was consistent with these reports, confirming supplemental feeding of QZGSP to perinatal sows could improve the liver health and alleviate oxidative damage to the livers.

Supplemental feeding of QZGSP to perinatal sows also resulted in improvements in the postpartum immunity of sows. We examined immunoglobulin and cytokine levels in sow serum, colostrum and milk to assess maternal antibody levels and transfer of passive immunity. As the main reactive substances of the humoral immune response, Ig levels can reflect the body’s immune function accurately (63). Previous research has shown that supplementing the diet with APS significantly increases levels of IgG and IgM in sow colostrum (64). Supplemental feeding of soybean isoflavone and astragalus polysaccharide mixture to lactating sows significantly increased the levels of IgA in colostrum (35). Hao et al. (32) demonstrated a significant enhancement in serum levels of IgA and IgG when 15 g/kg Astragalus powder was included in the diet of fattening lambs. Similarly, Xia et al.’s research revealed that Astragalus polysaccharide dietary supplementation effectively stimulated the secretion of IgA and IgG in the serum of weaned rabbits (65). The mice immunized with PCV2 antigen adsorbed *A. sinensis* polysaccharide (ASP)—Poly (lactic-co-glycolic acid) (PLGA)—polyethylenimine (PEI) nanoparticles significantly enhanced PCV2-specific IgG immune response (66). Our findings are consistent with previous studies, indicating that supplemental feeding of QZGSP to perinatal sows significantly enhances the synthesis of immunoglobulins. From the role of cytokines in inflammation, certain cytokines (e.g., TNF-α and IL-1β) are commonly referred to as pro-inflammatory cytokines due to their involvement in promoting inflammation. Conversely, other cytokines (such as IL-10 and IL-4) are known as anti-inflammatory cytokines due to their ability to inhibit inflammation (67). Flavonoids are the important active healthcare components in *A. membranaceus* (68). Dietary supplementation of a citrus total flavonoid extract in lactating dairy cows linearly decreased serum TNF-α and IL-1β levels (69). Feeding crude extracts of *A. macrocephala* and Glycyrrhiza Radix to a chicken model of oxidative stress significantly reduced the levels of TNF-α, while increasing the levels of IL-10 and IL-4 in serum (70). Danggui Buxue decoction (DBD) significantly reduced the levels of TNF-α, IL-1β in serum from GK rats with type 2 diabetes (71). Our results are consistent with previous reports, and the general trend of changes in sow cytokine levels aligns with the expectation that QZGSP can significantly enhance immunity in sows and outperform the other test groups. This suggests that supplementing periparturient sows with QZGSP can effectively regulate their cytokine secretion and enhance their cellular immunity level. In conclusion, supplementing periparturient sows with 2 kg/t or 3 kg/t of QZGSP significantly enhances their immunity after parturition. However, due to the influence of the African swine fever epidemic, there is a scarcity of literature related to the effect of Chinese herbs contained in QZGSP on sow health and production performance. Therefore, comprehensive comparisons cannot be made. Nevertheless, this remains the direction for future efforts by our group. Further in-depth research is still needed to investigate the specific mechanism by which periparturient sows’ immunity is enhanced through supplemental feeding of QZGSP.

Breast milk serve as the main nutritional source for newborn piglets, providing essential carbohydrates, lipids, and proteins (immunoglobulins) that are crucial for both nutrition and immune function (72). Therefore, the composition of breast milk can be used as an indicator of the nutritional levels of lactating sows. Previous research has shown that APS improves the components in sow colostrum and/or milk (64). Supplemental feeding of a mixture of soy isoflavones and *A.* polysaccharides increased lactation performance in sows, but did not significantly improve colostrum composition (35). Our findings revealed that supplementing perinatal sows with 2 kg/t and 3 kg/t of QZGSP significantly increases fat and protein contents in colostrum and milk compared to other experimental groups. This finding is consistent with previous reports, which suggests that supplemental feeding of QZGSP to periparturient sows significantly improves the conventional nutrient composition of sow milk. Breast milk not only plays a crucial role in nourishing and protecting newborn mammals but also shapes the development of their intestinal microbiota. α- and β-diversity are mainly used to assess the diversity of microflora. Our results shown that the diversity index (including Chao1 and Ace) of milk microbiota in sows from TRT3 and TRT4 groups was significantly higher than that in TRT2 group on day 21 of lactation, while there was no difference in the α-diversity of colostrum microbiota among different groups, which was inconsistent with Chen et al.’s research (73), and may be due to herd breed, rearing environment, and dietary addition of drugs. PCoA analysis based on unweighted_unifrac distance revealed significant differences was observed in colostrum and milk flora between the CON group and the TRT3 and TRT4 groups on days 0 and 21 of lactation, as well as a significant difference in milk flora between TRT1 and TRT4 groups on day 11 of lactation. These findings suggest that treatment with TRT3 and TRT4 can effectively modulate both the species composition and abundance of major colostrum and milk flora. Overall, supplemental feeding of QZGSP to perinatal sows could improve the diversity of microflora in sows’ colostrum and milk.

Supplemental feeding of QZGSP to perinatal sows resulted in changes in the microbiota structure in sow’s colostrum and milk. Specifically, the relative abundance of Escherichia-Shigella, Staphylococcus, and Streptococcus was decreased in the TRT3 and/or TRT4 groups on day 0 of lactation compared to other experimental groups. Species difference analysis showed that at the phylum level, the abundance of Bacteroidota, Desulfobacterota, and Actinobacteriota in the TRT4 group was significantly higher than that in the TRT1 group on days 0 and day 21 of lactation. At the genus level, the abundance of Rothia in the TRT4 group was significantly higher than that in the TRT1 group on day 21 of lactation. Previous reported that a combination herbal medicine with *A. membranaceus* and *A. macrocephala* Koidz. as the main components significantly reduced the relative abundance of Escherichia-Shigella in the intestinal tract of broiler chickens (74). Feeding fermented Chinese medicine to lambs can regulate the sensitivity of intestinal flora to pathogenic microorganisms such as *Escherichia coli* and *Staphylococcus aureus*, reducing the relative abundance of harmful bacteria (75). Polysaccharides derived from *A. membranaceus* and Glycyrrhiza uralensis significantly decreased the abundance of *Bacteroidetes* and *Desulfovibrio* in broilers (76). Oral administration of *A. sinensis* polysaccharide (ASP) reduces mammary inflammation and damage to the blood-milk barrier (BMB) induced by *Staphylococcus aureus* in mice, primarily through the modulation of intestinal flora (77). The aqueous extract of *P. lactiflora* altered the abundance of intestinal microbiota in mice with dextran sodium sulfate-induced colitis and reduced the levels of Bacteroides and Escherichia-Shigella (23). These results are consistent with our original study reporting (78), whereas maternal gut bacteria can enter breast milk via the entero-mammary pathway, this in turn affects neonatal intestinal colonization and immune system maturation (79). In the present experiment, supplemental feeding of 2 kg/t and/or 3 kg/t QZGSP to periparturient sows significantly decreased the relative abundance of harmful bacteria and increased the relative abundance of beneficial bacteria in sow colostrum and breast milk, suggesting that supplemental feeding of QZGSP to periparturient sows may affect the composition and structure of the microflora of sow colostrum and breast milk through modulation of the intestinal flora. In general, an increase in probiotic bacteria and a decrease in pathogenic bacteria in animal milk may contribute to improved milk quality in sows. It is reasonable to assume that the changes in the associated bacteria were caused by the herbal complexes. In conclusion, herbs play a very important role in regulating the microflora balance of breast milk during the perinatal period. Meanwhile, herbs, as alternatives to antibiotics and as feed additives, have great potential to improve the reproductive performance, immunity and breast milk quality of periparturient sows, but it is still important to note that the composition of herbs is very complex, and their mechanism of action needs to be investigated in detail in order to ensure their successful application in periparturient sows’ diets.

## 5 Conclusion

In summary, supplemental feeding of QZGSP to perinatal sows significantly improved reproductive performance, immunity, and breast milk quality in sows. The optimum dosage of QZGSP in this study was 2 kg/t.

## Data Availability

The datasets presented in this study can be found in online repositories. The names of the repository/repositories and accession number(s) can be found at: NCBI, PRJNA1123000.

## Footnotes

^1^ http://www.omicsmart.com
